# Different profiles of chemokines, cytokines and cell growth factors in plasma samples from patients with leprosy, leprosy reactions and households contacts

**DOI:** 10.1590/0074-02760230129

**Published:** 2024-02-19

**Authors:** Jairo Campos de Carvalho, Marcelo Antônio Pascoal-Xavier, Marcelo Grossi Araújo, Andrea Teixeira-Carvalho, Olindo Assis Martins-Filho, Vanessa Peruhype-Magalhães, Jordana Grazziela Alves Coelho-dos-Reis, Márcio Sobreira Silva Araújo

**Affiliations:** 1 Fundação Oswaldo Cruz-Fiocruz, Instituto René Rachou, Grupo Integrado de Pesquisas em Biomarcadores, Belo Horizonte, MG, Brasil; 2 Fundação Hospitalar do Estado de Minas Gerais, Belo Horizonte, MG, Brasil; 3 Universidade Federal de Minas Gerais, Faculdade de Medicina, Departamento de Anatomia Patológica e Medicina Legal, Belo Horizonte, MG, Brasil; 4 Serviço de Dermatologia do Hospital das Clínicas da Universidade Federal de Minas Gerais, Belo Horizonte, MG, Brasil; 5 Universidade Federal de Minas Gerais, Instituto de Ciências Biológicas, Departamento de Microbiologia, Laboratório de Virologia Básica e Aplicada, Belo Horizonte, MG, Brasil

**Keywords:** leprosy, cytokine, chemokines, Luminex

## Abstract

**BACKGROUND:**

Leprosy is a highly neglected disease that is considered a serious public health problem in many countries. This illness is characterised by a variety of clinical and histopathological manifestations that are related to the patient immune response.

**OBJECTIVES:**

This work aimed evaluate the profile of circulating immune mediators in the plasma from patients classified clinically as paucibacillary (PB), multibacillary (MB), households contacts (HHC), type1 leprosy reaction (T1R), type2 leprosy reaction (T2R) and control individuals without medical history of leprosy (CTL).

**METHODS:**

To assessment of the plasma immune mediators was used multiplex microbeads immunoassay “Luminex”.

**FINDINGS:**

The results showed that patients (PB) had a regulatory-biased profile, while MB revealed a pro-inflammatory trend of highly expressed biomarkers. HHC display conspicuously increased levels in the plasma of the chemokines (CCL2, CCL3, CCL4, CCL5 and CXCL8), pro-inflammatory cytokines (IFN-γ,TNF and IL-1β), modulating cytokines (IL-9 and IL-1Ra) and growth factors (PDGF, G-CSF and IL-2). Interestingly, HHC displayed superior production of IFN-γ as compared to other leprosy groups, indicating a putative protective role for this cytokine during chronic *Mycobacterium leprae* exposure.

**MAIN CONCLUSION:**

Further investigations are currently underway to elucidate the potential of these mediators as biomarkers applicable to the diagnosis/prognosis of leprosy and also T1R and T2R leprosy reactions.

Leprosy is a contagious and chronic disease, potentially disabling and of great relevance to public health due to its high prevalence, related to low socioeconomic levels and poor hygiene.^1^

The main route of transmission of *Mycobacterium leprae*, occurs through the upper airways of a positive bacilliferous individual, untreated for a susceptible individual.^2^ ^,^ ^3^ ^,^ ^4^ ^,^ ^5^ After infection, the bacillus has access to the bloodstream and can reach the peripheral nerves and skin, but the infection can also involve the eyes, mucosa, upper respiratory tract, muscles, bones and testicles.^6^ ^,^ ^7^ ^,^ ^8^ The disease is manifested by skin lesions with altered sensitivity, thickening of peripheral nerves mainly in the limbs and is considered one of the main causes of physical disability due to neural involvement.^9^

The clinical and immunopathological manifestations associated with disease express a direct relationship with the inflammatory mediators present during the progression of the infectious process and determining the clinical forms of the disease.^10^ ^,^ ^11^ The paucibacillary (PB) form of leprosy is characterised in histopathology as it presents as tuberculoid granulomas, which in turn are related to a pro-inflammatory cellular immune response profile. In these lesions, the cytokine IL-12, IFN-γ and TNF acts on macrophages stimulating phagocytosis, the production of reactive oxygen and nitrogen reagents and the secretion of chemokine CXCL10.^6^ ^,^ ^12^ IL-2 together with the chemokines CCL2, CCL11 and CXCL10 activates CD4^+^ T lymphocytes, stimulating cell proliferation and promoting the maintenance of cytokines that will potentiate the effects of IFN-γ.^13^ ^,^ ^14^ ^,^ ^15^ ^,^ ^16^ ^,^ ^17^ Plasma levels of the chemokines CCL2, CCL3 and CCL11 can be detected in patients with leprosy lesions and remain unchanged after leprosy treatment.^18^ Interestingly, the chemokine CXCL10 was considered by Stefani et al.^17^ as a biomarker of leprosy reversal reaction, since serum levels are increased in relation to patients with leprosy, but without reaction. These same authors mention that IL-7 and PDGF represent potential biomarkers of erythema nodosum leprosum [type 2 leprosy reaction (T2R)]. Fiallo et al.^19^ found that the vascular endothelial growth factor (VEGF) and its KDR receptor are highly expressed in the granuloma cells of patients with reversal reaction, this may not only be relevant during hyperpermeability and differentiation of mononuclear cells but may also be involved in the beginning of the reversal reaction, when dendritic cells are activated in response to antigenic stimulation.

The multibacillary (MB) form of leprosy is characterised by a predominantly modulating immune response. The progressive and diffuse infiltration in the skin, mucous membranes, upper airways, eyes, testicles and nerves is very characteristic in this pole of the disease, reflecting the effects of a deficient cellular immune response.^20^ IL-4, for example, suppresses the activity of macrophages, blocking the action of TNF, IL-2, IFN-γ and IL-1β.^21^ ^,^ ^22^ The *M. leprae* bacillus is able to stimulate the production of IL-10 and TGF-β, which in turn will inhibit nitric oxide and TNF production and expression of major histocompatibility complex (MHC) and co-stimulatory molecules CD80 / CD86 by antigen presenting cells, preventing the production of pro-inflammatory cytokines by CD4^+^ T cells.^22^ ^,^ ^23^ ^,^ ^24^ ^,^ ^25^

In addition to these two types of immune response, recent studies have shown the presence of Th9 immune response in leprosy. In the PB form, IL-9 has a pro-inflammatory action because it inhibits the action of IL-10. In the MB form, the expression of IL-9 has a different character, being able to inhibit the production of IL-4, IFN-γ and TNF. In this type of response, IL-10 contributes to the development of an immunomodulatory response.^26^ ^,^ ^27^

Another response from the sub-population of T lymphocytes, T helper 17 (Th17), has been found in patients with tuberculoid leprosy and the elevation of IL-17 contributes to the recruitment of inflammatory cells (mainly neutrophils), activation of endothelial cells and maintenance of the chronic inflammatory process by inducing the production of TNF, IL-6 and iNOS.^28^ Th17 cells have been associated with inflammatory processes due to demyelination caused by damage to peripheral nerves. In stabilising the inflammatory process, IL-17A negatively regulates the production of neural growth factor (NGF) and its receptor in the tuberculoid and lepromatous clinical forms, contributing to the most severe form of the disease.^11^ Saini et al.^29^ described that the Th17 response, through the production of the cytokines IL-17A, IL-17F and IL-21, are associated with the processes of reversal reaction [type 1 leprosy reaction (T1R)].

In spite of the large number of studies showing the importance of cytokines and chemokines during chronic leprosy, few studies have shown a broad analysis of these mediators measured by novel high throughput technologies that allow for measuring each cytokine on the same sample. In addition, few studies have explored the inter-relationship amongst these mediators, according to the clinical forms of leprosy.

Therefore, the aim of this study was to evaluate the relationship amongst broad spectrum of cytokines, chemokines and growth factors in the plasma of leprosy patients in order to outline the unprofile of immunological biomarkers associated with different clinical forms of leprosy, leprosy reaction and households contacts using cutting edge high-throughput screening technology.

## MATERIALS AND METHODS

*Study population* - The present investigation was carried out as observational study comprising of a non-probabilistic convenience sampling. The study population comprised a total of 40 leprosy patients with no history of multidrug therapy and without records of using immunosuppressive treatment. Leprosy patients were enrolled upon routine medical appointments at the Dermatology Clinic of Faculdade de Medicina, Universidade Federal de Minas Gerais. The inclusion criteria comprised volunteers of both sexes, older than 18 years with no previous selection based on ethnic features, educational level or economical class who agreed to participate in the study. Patients were further categorised according to the operational classification into subgroups, including tuberculoid and borderline-tuberculoid leprosy patients unified in the PB subgroup (PB, n = 14) and patients with the borderline-borderline, borderline-lepromatous forms unified in the MB subgroup (MB, n = 13). Leprosy patients presenting reversal reaction were included in the T1R subgroup (T1R, n = 12) and patients with clinical signs of erythema nodosum leprosum comprised the T2R subgroup (T2R, n = 11). In the T1R group, 11/12 (92%) were PB patients, while in the TR2 group all patients (11/11) were MB. Two control groups were included in the present study, comprising healthy subjects living in endemic areas for leprosy and residents in non-endemic areas, both subgroups from Minas Gerais State, Brazil. A group of household contacts (HHC), comprising healthy volunteers companions of leprosy patient were selected using the same inclusion criteria and included subjects with no clinical signs of leprosy contacted at the outpatient unit (HHC, n = 6) at the time of leprosy patient selection. The HHC group were composed by 5/6 (83%) of HHC of MB leprosy patients. A control group of healthy volunteers with no clinical history of leprosy (control group - CTL, n = 18) was composed by volunteers living at the metropolitan area of Belo Horizonte, Minas Gerais, Brazil.

The study protocol was submitted and approved by the Research Ethics Committee of the Instituto René Rachou - FIOCRUZ-Minas (CAAE: 77737317.1.0000.5149) and Federal University of Minas Gerais (COEP/UFMG, CAAE - 38.544.914.0.3001.5149). The Table summarises the major demographical and clinical features of the study groups.

**TABLE:** Demographic and clinic aspects of study population

Parameters	CTL	HHC	PB	MB	T1R	T2R
Participants (n)	18	6	14	13	12	11
Sex						
Man (%)	8 (44.4)	2 (33.3)	6 (42.9)	7 (53.8)	9 (75.0)	10 (90.9)
Women (%)	10 (55.6)	4 (66.7)	8 (57.1)	6 (46.2)	3 (25)	1 (9.1)
Age, median (min-max)	38 (22-56)	37 (21-53)	38 (19-47)	48 (34-65)	49 (21-62)	45 (27-64)
BI, median (min-max)	-	-	0 (0-0)	2.8 (2.5-5.5)	0 (0-3.5)	3.0 (2.8-5.0)
Number of lesions, median (min-max)	-	-	3.5 (0-20)	20 (1-20)	20 (1-30)	20 (20-30)
Number of affected nerves, median (min-max)	-	-	0 (0-3)	1.5 (0-3)	2 (0-3)	2 (0-2)

CTL: control group of subjects without medical history of leprosy; HHC: households contacts group; PB: patients with paucibacillary leprosy; MB: patients with multibacillary leprosy; T1R: patients with type 1 leprosy reaction (Reversal reaction); T2R: patients with type 2 leprosy reaction (Erythema nodosum leprosum); BI: bacilloscopic index.

*Analysis of chemokines, cytokines and cell growth factors in plasma samples employing multiplex microbeads immunoassay* - Peripheral blood samples were collected in vacuum tubes containing sodium heparin as anticoagulant and the plasma samples collected were stored at -80^o^C until the use. The levels of chemokines (IL-8 = CXCL8, eotaxin = CCL11, macrophage inflammatory protein 1α = CCL-3, CCL-4, monocyte chemotactic protein 1 = CCL-2, RANTES = CCL-5 and IFN-inducible protein-10 = CXCL10), cytokines (IL-1β, IL-6, TNF, IL-12, IFN-γ, IL-15, IL-17, IL-1Ra, IL-4, IL-5, IL-9, IL-10, IL-13) and growth factors (basic fibroblast growth factor = FGF-basic, platelet-derived growth factor = PDGF, vascular endothelial growth factor = VEGF, granulocyte colony-stimulating factor = G-CSF, granulocyte-macrophage colony-stimulating factor = GM-CSF, IL-2 and IL-7) and were evaluated by “Bio-Plex Pro Human Cytokine 27-Plex” system (Bio-Rad Laboratories, California, USA), according to the manufacturer’s instructions.

The plasma samples were incubated with capture beads and run-in duplicate to measure the plasma biomarkers concentrations. One lot of the reagents was used for the entire study (plate and standards). A minimum of 50 beads per biomarker was acquired. The median fluorescence intensities were measured using a Bio-Plex 200 instrument using Bio-Plex Manager software version 6.0 (Bio-Rad Laboratories, California, USA). Standard curves for each biomarker were generated using the premixed lyophilised standards provided in the kits. Serial 4-fold dilutions of the standards were run to generate a 9-standard concentration set, and the diluent alone was used as blank. The concentrations of soluble mediators were determined from the standard curve using a 5-parameter logistic fit curve to transform the mean fluorescence intensities into concentrations. The results were expressed as pg/mL.

Statistical analysis

*Descriptive data analysis* - Statistical analysis was carried out to assess the profile of chemokines, cytokines and growth factors were performed using the GraphPad Prism 5.0 software (GraphPad San Diego, CA, USA). The normality test indicated non-parametric data distribution. Thus, the Kruskal-Wallis test was applied, followed by the Dunn´s multiple comparison test. Statistically significant differences were considered when p < 0.05.

*Biomarker signatures* - The plasma biomarker ascendant signatures were assembled as previously reported by Costa-Pereira et al.^30^ To perform the ascending signature analysis, the global median values of each variable (chemokines, cytokines, and growth factors) were calculated and used to classify individuals with low and high plasma concentration, when they present results below or above the global median, respectively. The cut-off global median values were determined considering the values of all study groups: CTL, HHC, patients PB leprosy, MB patients, patients with T1R and T2R. Thereafter, the number of individuals classified as high producers was used to calculate the frequency of subjects with plasma concentration above the cut-off. This frequency was then placed in ascending order to select the most relevant biomarkers (frequency > 50%). The ascending curves generated for each group of individuals were overlayed for comparative analysis.

*Correlation network of biomarkers* - The correlation networks were assembled to evaluate the multiple associations amongst the chemokines/cytokines/growth factors within each leprosy group (PB, MB, T1R, T2R, HHC) and compare with the CTL. Similar to that described by Garcia et al.,^31^ the association between the quantitative levels of chemokines, cytokines and growth factors were determined by the Spearman and Pearson correlation coefficient for parametric and nonparametric data, respectively, using GraphPad Prism 5.0 software (San Diego, USA). Statistical significance was considered only at p < 0.05. The r values of significant correlations were used to create correlation matrix using the Microsoft Office Excel™ Software (program 2010). These matrices containing the correlation indices characterised as positive or negative were used for constructing the correlation networks using the open-acess the Cytoscape™ program (version 3.6.1). The biomarker networks were constructed using circular layout with nodes representing for each soluble mediator as follows: chemokine (blue nodes), pro-inflamatory cytokines (red nodes), regulatory cytokines (green nodes) and growth factor (yellow nodes).

## RESULTS

**Fig. 1:**
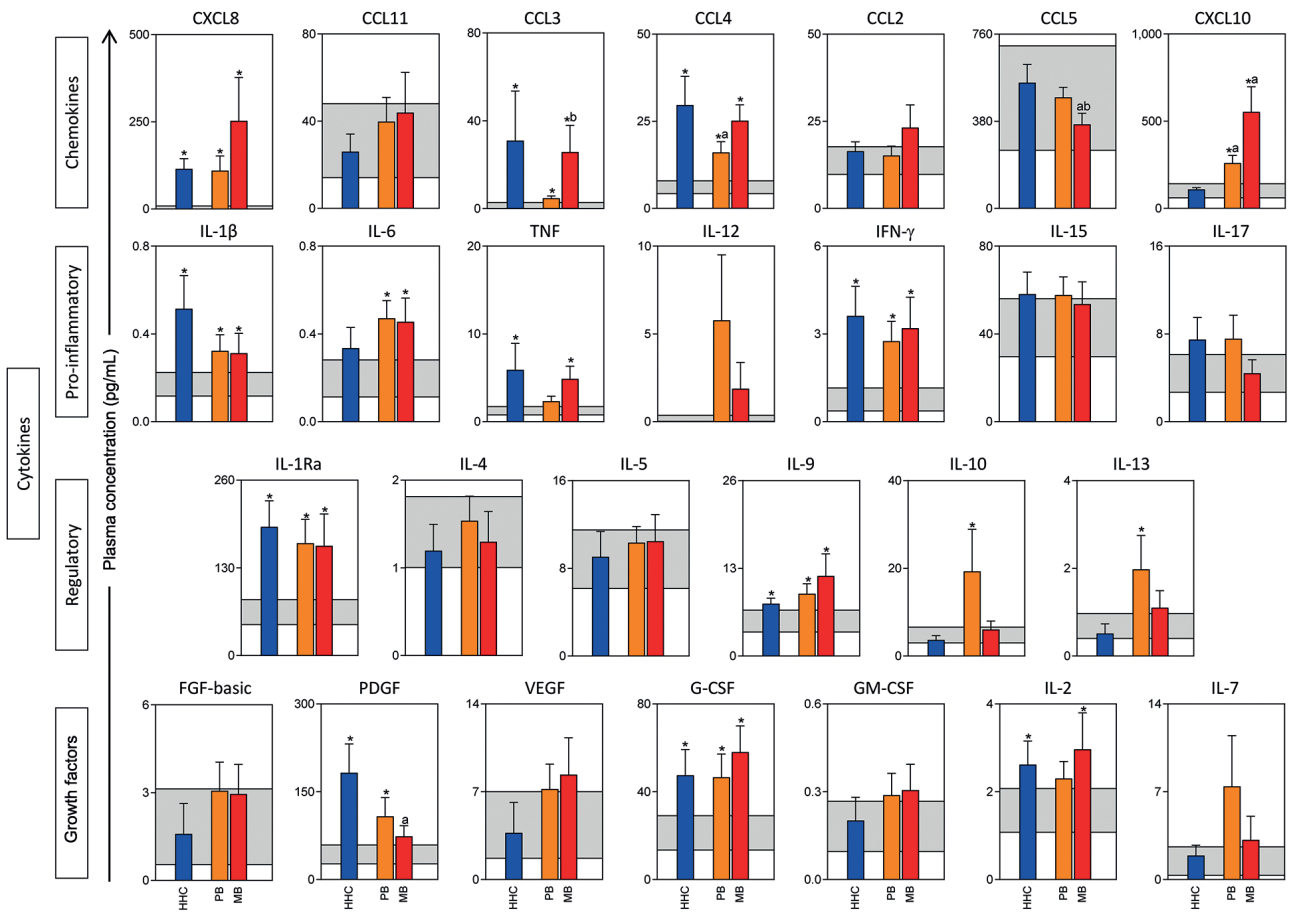
plasma concentration of chemokines, pro-inflammatory/regulatory cytokines and growth factors in patients with paucibacillary (PB), multibacillary (MB) leprosy and household contacts (HHC). Plasma concentrations of chemokines, proinflammatory/regulatory cytokines and growth factors are shown in picograms per milliliter (pg/mL) in bar graphs highlighting the mean and standard error. The group of HHC living in endemic areas is represented by blue bar, the PB group by the orange bar and the MB group by the red bar. The gray background on each graph represents the 95% confidence interval (CI) of values observed in the plasma samples from the healthy control group living (CTL) in non-endemic areas. The significant differences (p < 0.05) for comparisons with the CTL are represented by the asterisk (*) and the significant differences (p < 0.05) for comparisons with HHC and PB groups are represented by the letter “a” and “b”, respectively.

*A pro-inflammatory response is remarkable in PB, MB leprosy and HHC* - In order to evaluate the profile of circulating immunological mediators associated with leprosy, 27 analyses were simultaneously quantified in the plasma of leprosy patients and HHC living in endemic areas and healthy controls from non-endemic areas, including: chemokines (CXCL8, CCL11, CCL3, CCL4, CCL2, CCL5 and CXCL10), pro-inflammatory cytokines (IL-1β, IL-6, TNF, IL-12, IFN-γ, IL-15 and IL-17), regulatory cytokines (IL-1Ra, IL-4, IL-5, IL-9, IL-10 and IL- 13) and growth factors (FGF-basic, PDGF, VEGF, GM-CSF, G-CSF, IL-2 and IL-7). Leprosy patients were classified as PB, MB leprosy and the soluble mediator profile compared with that observed for healthy controls (HHC and CTL). The results are shown in Fig. 1. The analyses of the results demonstrated that higher plasma concentrations of chemotactic factors associated with migration of neutrophils, macrophages, monocytes, and NK cells (CXCL8, CCL3, CCL4 and CXCL10) were observed for PB and MB patients as compared to the healthy CTL, living in non-endemic areas. In addition, data showed that pro-inflammatory cytokines (IL-1β, IL-6 and IFN-γ) presented an increased concentration of in the plasma of PB and MB as compared to CTL group. Interestingly, our data demonstrated increased levels of IL-1Ra and IL-9 as well as G-CSF in plasma samples of PB and MB. However, the results suggest a modulatory immune profile in PB group characterised by increased IL-10 and IL-13 production as compared to CTL. On the other hand, MB patients presented an elevated plasma concentration of TNF, reinforcing the pro-inflammatory profile, as compared to CTL group. Moreover, data analysis demonstrated higher CCL3 and lower CCL5 plasma levels in MB as compared to PB patients (Fig. 1).

It is important to observe that differential biomarker profiles were also observed for HHC individuals as compared to CTL group. Our data clearly showed that HHC presented a similar immunological profile to the one observed in PB and especially in MB patients. These results showed increase levels of CXCL8, CCL3 and CCL4, pro-inflammatory cytokines such as IL-1β, TNF and IFN-γ as well as IL-1Ra, IL-9, PDGF, G-CSF and IL-2 in HHC group as compared to CTL (Fig. 1).

**Fig 2:**
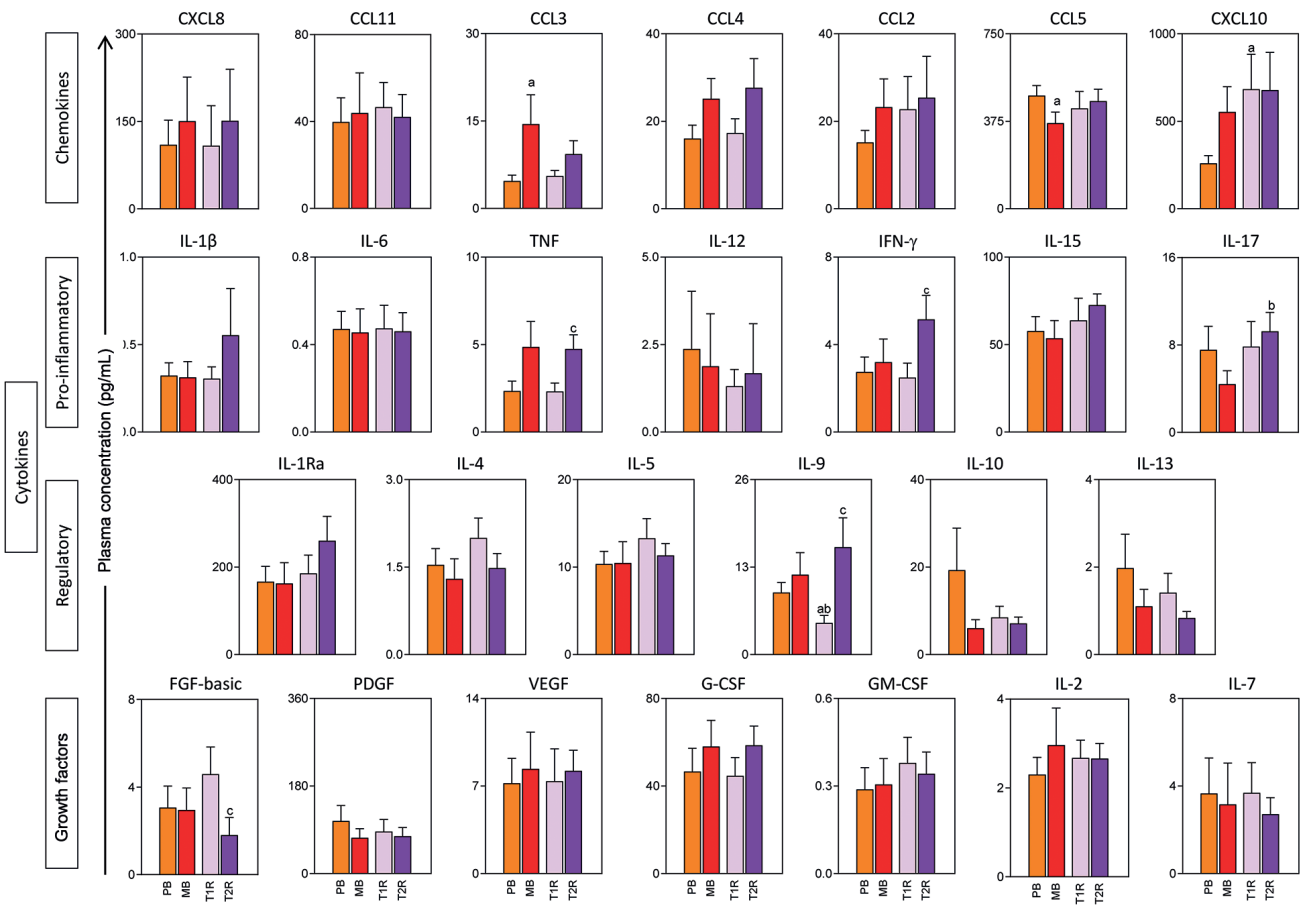
plasma concentration of chemokines, proinflammatory/regulatory cytokines, and growth factors in patients with paucibacillary (PB), multibacillary (MB) leprosy and leprosy reactions type 1 (T1R) and type 2 (T2R). Plasma concentrations of chemokines, proinflammatory/regulatory cytokines and growth factors rates are shown in picograms per milliliters (pg/mL) in bar graphs highlighting the mean and standard error. The PB group is represented by the orange bar, the MB group by the red bar, the T1R group by the light purple bar and the T2R group by the dark purple bar. The significant differences (p < 0.05) for comparisons with the PB, MB and T1R groups are represented by the letters “a”, “b” and “c”, respectively.

*T2R leprosy reaction group is characterised by intense systemic inflammation mediated by TNF, IFN-γ, IL-9 and IL-17* - The analysis of the results for the concentration of immunological mediators in the plasma samples from PB and MB leprosy was carried out in comparison with T1R and T2R leprosy reaction and data are shown in Fig. 2. This data analysis strategy aims at identifying specificities of the acute inflammatory reactions (T1R and T2R) observed in leprosy.

The results demonstrated that differences between T1R and T2R that stand out in the comparative analysis suggest a very intense inflammatory reaction in T2R group, including higher levels of TNF, IFN-γ, IL-9 and IL-17 by T2R as compared to T1R (Fig. 2).

**Fig. 3:**
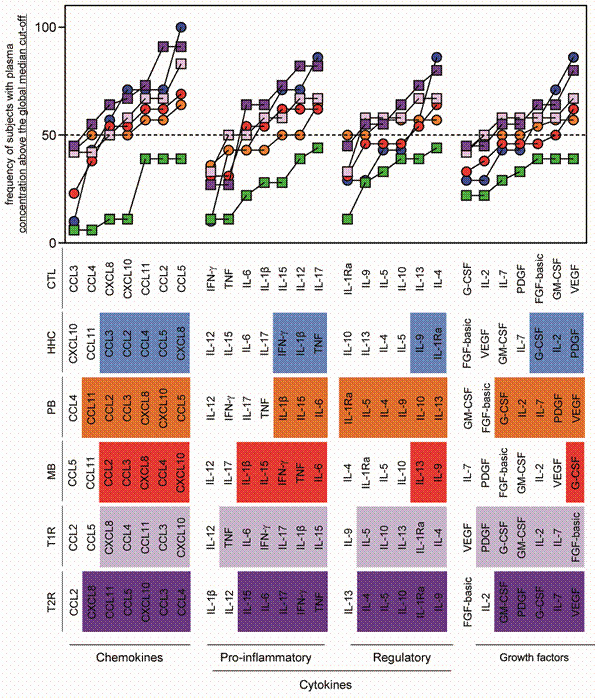
comparative analysis of ascending signatures of plasma biomarkers in patients with paucibacillary (PB), multibacillary (MB) leprosy, type 1 (T1R) and type 2 leprosy reactions (T2R) and households contacts (HHC). The panoramic profile of biomarkers (chemokines, pro-inflammatory/regulatory cytokines and growth factors) of each individual, in each group, is demonstrated by diagrams. The global median was the cut-off point for the segregation of individuals with low plasma concentration from biomarkers of individuals with high plasma concentration. The signature of plasma biomarkers in leprosy patients classified as PB group (orange circles), MB group (red circles), T1R group (light purple squares), T2R group (dark purple squares), healthy control groups (CTL): HHC living in endemic areas (blue circles) and CTL living in non-endemic areas (green squares) are presented as overlapping ascending signatures for comparative analysis of the profile of biomarkers between the study groups. The dotted line highlights the 50th percentile of the frequency of subjects with plasma concentration above the global median cut-off. The biomarkers presenting more than 50% of the subjects above the cut-off are highlighted by colour backgrounds, corresponding to each study group.

*Categorical descriptive analysis reinforce the distinct immunological profile presented by leprosy patients* - Another strategy used to characterise and compare the immune response of patients with leprosy was the signature of immunological biomarkers represented by the ascendant biomarker curves as shown in Fig. 3. The comparative analysis of the immune mediator signatures amongst the study groups (HHC, PB, MB, T1R and T2R) was performed by overlapping the ascendant signatures of each group as described in material and methods. Using this categorical descriptive approach, it was possible to identify biomarkers observed in more than 50% of the individuals on each group (Fig. 3). Overall, the healthy CTL did not present biomarkers above the 50th percentile.

Moreover, this categorical analysis clearly shows that PB patients have a balanced immunological profile, characterised by the presence of pro-inflammatory cytokines (IL-1β, IL-15 and IL-6) and regulatory cytokines (IL-1Ra, IL-5, IL-4, IL-9, IL-10 and IL-13), above the 50th percentile threshold. On the other hand, data reinforces the highly pro-inflammatory profile of MB patients, characterised by the presence of IL-1β, IL-15, IFN-γ, TNF and IL-6 cytokines, with minor regulatory cytokines (IL-13 and IL-9) above the 50th percentile.

It is very important to highlight the profile of the ascendant biomarker curves presented by individuals of the HHC group. The categorical analysis clearly shows the similarities already described between the HHC and MB groups, suggesting that a responsive and exuberantly altered immune system is present in those patients as a marker of *M. leprae* exposure.

Comparative analysis between patients with T1R and T2R showed that both types of leprosy reaction display higher frequency of patients with altered production of several biomarkers, suggesting intense immune activation in these cases. In addition, the data confirmed similarities between T1R and T2R patients as described previously. The data also showed a mixed pro-inflammatory/regulatory profile, demonstrating similarities with both MB and PB groups (Fig. 3).

**Fig. 4:**
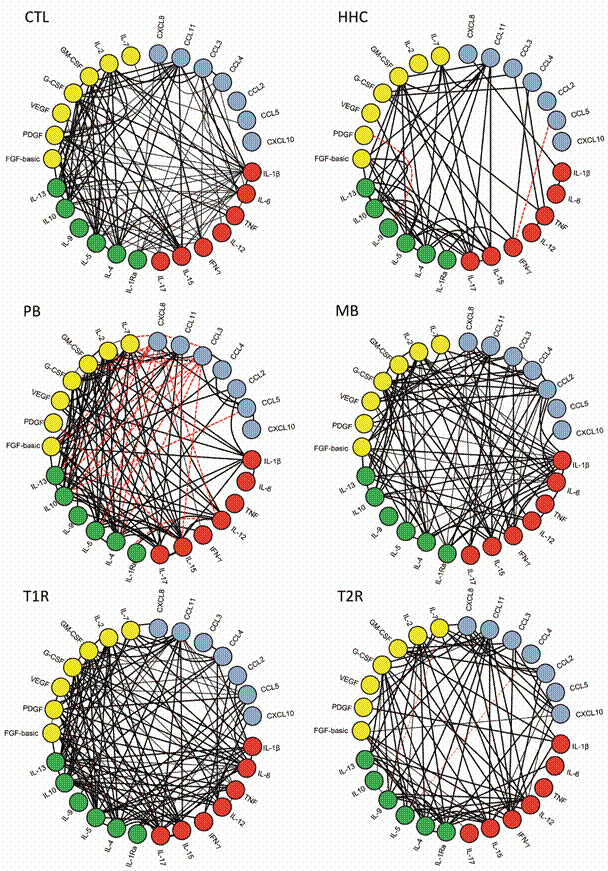
correlation networks between different plasma biomarkers in patients with paucibacillary (PB), multibacillary (MB) leprosy, type 1 (T1R) and type 2 leprosy reactions (T2R) and households contacts (HHC). Correlation networks of chemokines (blue circle), proinflammatory cytokines (red circle), regulatory cytokines (green circle) and growth factors (yellow circle) were assembled for each study group: healthy controls living (CTL) in non-endemic areas, HHC living in endemic areas, PB, MB, T1R) and T2R. The black lines show positive correlations between the biomarkers and the red dotted lines underscore the negative correlations.

*Distinct biomarker networks are present in PB, MB leprosy, T1R and T2R patients and HHC* - In order to understand the interconnection and relationship between the plentiful groups analysed, correlations analysis was carried out to construct an integrative network of immunological biomarkers (Fig. 4). The integrative networks allowed for a panoramic view of data and its association in each leprosy group.

The exploratory analysis demonstrated that in the CTL, a complex and positive correlation network was present, especially in pro-inflammatory/regulatory cytokines and growth factors axis. On the other hand, in the interactive network of the HHC group, was possible to observe that the level of complexity decreased, and many correlations were lost as compared to CTL, especially those involving chemokines and pro-inflammatory cytokines.

In the analysis of the biomarker network of PB group, it was possible to visualise an expressive number of correlations between growth factors and regulatory cytokines. The most striking feature of the network generated for this group was the presence of many negative correlations between regulatory cytokines and chemokines. Another characteristic observed was the loss of correlations between pro-inflammatory cytokines and other biomarkers.

The correlation networks analysis showed that MB patients have a complex and different interaction profile from the presented by PB group. The data demonstrated an intense correlation for the pro-inflammatory cytokines and chemokines axis. Greater involvement of the growth factors can also be observed for this group. In addition, negative correlations were not observed between the biomarkers in this group.

The interactive biomarkers network displayed for the group of T1R was more complex and more concentrated, with marked involvement of growth factors, regulatory cytokines, and pro-inflammatory cytokines. Conversely, the level of complexity decreased in T2R network. The network of the T2R group had lower number of correlations, although it involved all the variety of biomarkers tested. In this group, the presence of some negative correlations was also observed, which were absent in the T1R group (Fig. 4).

## DISCUSSION

Leprosy is an infectious, granulomatous and chronic disease with high disabling power that can lead to physical disabilities and deformities due to its potential to cause neural injuries.^4^ It is a spectral disease, clinical manifestations are directly linked to the individual’s immune response.^32^ ^,^ ^33^ ^,^ ^34^ ^,^ ^35^ During the chronic course of the disease, acute inflammatory manifestations can occur and evolve clinically to neural and skin lesions named leprosy reactions. These reactions are complications of the disease due to changes in the host’s immune balance against the bacillus and are directly involved with the injuries and morbidities. Understanding the immunological events associated with clinical manifestations is important to support the establishment of new strategies for the clinical management of leprosy patients and to monitor the progression of the disease and the development of leprosy reactions.^36^

In this regard and in agreement with present findings, the work by Kirkaldy et al.,^37^ using the immunohistochemistry technique, detected the expression of the chemokines CCL2, CCL5 and CXCL8 in lesions of different clinical forms of leprosy, but did not differentiate the intensity of staining in the tissues. Hasan et al.,^38^ found a reduction in CCL2 levels and an increase in circulating CCL5 levels in leprosy patients when compared to individuals in the control group. These results corroborate in part our data, that demonstrated that plasma concentration of CCL2 was increased and CCL5 decreased in patients with MB leprosy in relation to the HHC and PB groups. Similarly, elevated levels of CCL3, CCL4, CXCL8 and CXCL10 were observed in PB and MB patients. Mendonça et al.^16^ and Queiroz et al.,^39^ also observed that PB and MB patients had increased plasma concentrations of CCL2, CCL3, CCL11, CXCL8, CXCL9 and CXCL10 compared to the CTL. Specifically, our data on CXCL10 also showed that patients with T1R presented increase in plasma levels when compared to the PB group. The increase in CXCL10 levels in peripheral blood has been associated with neuritis during leprosy reactions.^17^ ^,^ ^40^

In leprosy patients, the production profile of pro-inflammatory or regulatory cytokines determines the clinical manifestation of the disease,^41^ however, measuring cytokines is yet to be of clinical use. When evaluating the expression of pro-inflammatory cytokines, it was observed that IL-1β and IFN-γ were increased in the PB and MB groups. In the literature, pro-inflammatory cytokines have been described as essential in the elimination of the bacillus, as they are able to induce the cellular immune response and stimulate the expression of nitric oxide.^42^ ^,^ ^43^ ^,^ ^44^ ^,^ ^45^ On the other hand, at high levels, they can also be involved in the process of inflammation of the nerves causing neuritis, present in leprosy reactions.^46^ In our study, we also observed an increase, especially of IFN-γ, in patients with T2R. In addition, we also observed an increase in TNF levels in the MB group and in T2R patients. It is known that this cytokine has ability to promote the activation of macrophages and potentiate the effects of IFN-γ.^12^ In immunohistochemistry analyses it has been described that TNF is expressed in epithelioid cell macrophages of PB and MB lesions and contributes to the formation of granulomas.^41^ ^,^ ^47^ TNF is able to regulate the expression of IL-6 and together enhance the inflammation process of nerve cells, causing neuritis in leprosy reactions.^40^ ^,^ ^44^ ^,^ ^48^

Leprosy reactions are episodes of acute inflammation and occur in response to the presence of *M. leprae* antigens. They affect a large proportion of leprosy patients and can occur before diagnosis, during and after treatment. In addition to the previously mentioned pro-inflammatory cytokines (IL-1β, IL-6, TNF and IFN-γ), it is also reported in other studies the increase in the expression of IL-17 in reaction episodes of leprosy, which may be present in these lesions even after treatment.^29^ ^,^ ^40^ ^,^ ^45^ ^,^ ^49^ Our results reinforce these findings, paving the road for more studies on the role of IL-17 in T2R.

In leprosy patients, the cytokine IL-10 is expressed in greater concentration in MB patients and in those with leprosy reaction, although it is also present in the plasma of PB patients. The action of IL-10 in leprosy lesions leads to the differentiation of regulatory CD4^+^ T cells, as well as regulatory B cells that produce high levels of IL-10. This cytokine acts on B cells inducing the antibody production, which potentiates the formation of immune complexes and maintains the suppression of the antigen-specific immune response, an important characteristic during T2R. This phenomenon may contribute to disease aggravation by favouring the multiplication of the bacillus.^25^ ^,^ ^41^ ^,^ ^43^ ^,^ ^50^ ^,^ ^51^ In the evaluation of IL-10 in the plasma from patients was observed that it was increased only in the group of patients with the PB form, differently from what has been described in the literature, which reports an increase mainly in MB forms and leprosy reactions. Likewise, other cytokine of profile regulatory the IL-13 was increased in the plasma of patients with PB leprosy. The work of Atkinson et al.^52^ confirms the presence of these two cytokines (IL-10 and IL-13) in the lesions caused by *M. leprae*. In addition, Cassirer-Costa et al.^44^ mentions that both cytokines play an important role after leprosy treatment, as they suppress the effects of IFN-γ in the lesions caused by the bacillus. Our findings suggest a possible and important role of IL-10 and IL-13 in regulate the pro-inflammatory immune response in PB patients, which differs from MB and T2R patients, who have an exacerbated pro-inflammatory response.

Another point that can help in understanding the role of IL-10 and IL-13 PB and MB leprosy and corroborate with our data, concerns the analysis of IL-9 levels in leprosy patients in this study. Our results point to increase in plasma levels of IL-9 in PB, but especially in MB and T2R. Although IL-9 has been known as a modulatory cytokine, Finiasz et al.,^53^ carried out a study based on peripheral blood mononuclear cells from non-sick donors, in culture under inactivated *M. leprae* stimulation that showed the pro-inflammatory role of IL-9. The authors observed that this cytokine was able to modulate the effects of IL-4, IL-10, and IL-13, acting in synergy with IFN-γ and IL-6. Thus, we believe that MB and T2R patients have an exacerbated pro-inflammatory response, and that IL-9 can modulate the production and function of IL-10 and IL-13 in these clinical conditions. In this same line of thought, IL-1Ra has been demonstrated as important, since it can be found in high plasma concentrations in lesions of patients with different clinical forms of leprosy.^54^ Thus, IL-1Ra was suggested by these authors as a possible biomarker to be used in the diagnosis of the disease. This cytokine has anti-inflammatory functions, as they are antagonists of the cytokine IL-1α and IL-1β, competing for the same receptor. In our study, the results also showed an increase in plasma levels of IL-1Ra in patients with PB and MB leprosy.

In our study, G-CSF and GM-CSF were increased in the leprosy patients (PB and MB). Also, GM-CSF shown increased in the plasma of individuals with T1R and T2R. These growth factors are directly related to cell proliferation, differentiation and migration of the neutrophils and macrophages and the stimulation of T cells in the inflammation site. Thus, these increase in leprosy patients, as observed in our results, can somehow be related to lesions both on the skin and at the neural level as described by Shi et al.^55^ and Zhang et al.^56^ who suggest that the high expression of these molecules in the tissue, can lead to serious damage.

Another factor of cell proliferation, the cytokine IL-2, has relevant role in the maturation of T lymphocytes and B lymphocytes. IL-2 act promoting the availability of cells with memory function and these cells may be directly involved in the control of bacillus replication.^57^ ^,^ ^58^ ^,^ ^59^ When assessing the plasma concentration of the IL-2, the results showed that individuals in the MB and HHC group had increased levels of IL-2 when compared to the CTL. Thus, it appears that MB and HHC individuals have an exacerbated inflammatory response, which could favour the control of bacillus multiplication, however, with tissue damage in MB cases, due to the reduced role of biomarkers associated with immune response modulation.

PDGF and VEGF play a significant role in the formation and growth of blood vessels and are highly expressed in cells of the mononuclear-macrophage lineage and in the vascular endothelium of granulomas. These molecules could be related to the formation of oedema in patients with T1R and of the vasculitis in patients with T2R.^17^ ^,^ ^19^ ^,^ ^60^ ^,^ ^61^ Our results are supported by the findings of these authors, being observed increases of PDGF and VEGF in the plasma of the patients with T1R and T2R when evaluated by signature curve.

Analyses of the signature curves and the network interactions between plasma mediators were employed to provide a panoramic view of the results and of the interaction between them in leprosy patients, these approaches allow a panoramic view of the results. Employing the ascending signature analysis curve of the groups of patients with leprosy (MB and PB) and leprosy reactions (T1R and T2R), could be observed an intense change in the expression of most of the biomarkers evaluated. This reinforces the idea that leprosy cannot be considered only as a skin disease, but rather a serious and systemic disease that can sometimes develop, causing eye, mucous membrane, orchitis, in addition to neural damage.^4^ ^,^ ^32^ When the 27 biomarkers were analysed by networks interaction, also was possible to observe that the disease significantly changed the connections between them differently in each group. In the PB group were seen the occurrence of many negative correlations, mainly between chemokines and regulatory cytokines as well as the loss of correlations between proinflammatory cytokines and other biomarkers. In the MB group, the number of correlations between inflammatory chemokines and cytokines increased in relation to the PB group, in addition to the greater involvement of chemokines. In the T1R group, the correlations were more complex and were more concentrated between cell growth factors and regulatory and pro-inflammatory cytokines. In the T2R group, fewer correlations were observed, in addition to some of them being negative. We believe that this variability shown in the soluble mediator networks between groups is in agreement with the complex clinical profile in which leprosy can manifest itself, from a small skin lesion to severe cases of the disease. It is important to highlight the predominantly pro-inflammatory profile of immunological biomarkers presented by individuals in the HHC group. Some authors suggest the inclusion of HHC in health programs, even recommending the use of chemoprophylaxis to prevent future clinical disease, damage and disability.^62^ ^,^ ^63^

The present study has some limitations. The non-probabilistic sampling approach used to enroll the participants suggest the relevance of further studies to validate these findings to a general statement applicable for populational basis. Moreover, the small number of samples re-enforce the relevance of developing additional investigations to further support these findings. Noteworthy in the knowledge that the circulating levels of soluble mediator may not reflect the local changes taking place in the skin of leprosy patients. Gaining more insight in parallel investigations of local and systemic immune response would contribute to achieve reliable biomarkers for clinical monitoring. Furthermore, a follow-up investigation of leprosy patients as well as HHC would also be relevant to verify whether a specific immunological biomarker would have a prognostic value to predict distinct clinical outcomes.

*In conclusion* - As leprosy is a spectral disease, which has a very challenging clinical diagnosis, we believe that the assessment of plasma levels of immunological mediators can be used in the future in methods for the diagnosis and/or prognosis of the disease, as well as for post-treatment clinical monitoring. The results observed in the interaction network showed that leprosy alters plasma levels of mediators in different ways and were clearly related to the complex clinical profile of leprosy manifestation. Regarding the HHC group, for the first-time detailed analysis assessing 27 markers of immune response, showed that these individuals present an inflammatory tendency profile with increased levels of chemokines (CXCL8, CCL3, CCL4, CCL2 and CCL5), pro-inflammatory cytokines (IL-1β, TNF and IFN-γ), modulating cytokines (IL-1Ra and IL-9) and growth factors (PDGF, G-CSF and IL-2).
